# Does medical education protect against nicotine dependence? A comparison of e-cigarette use among physiotherapy and humanities students

**DOI:** 10.3389/fpsyg.2026.1842763

**Published:** 2026-10-26

**Authors:** Józefa Dąbek, Magdalena Szynal, Patryk Brasse, Julia Żerdka, Oskar Sierka, Edyta Sierka

**Affiliations:** 1 Department of Cardiology, Faculty of Health Sciences in Katowice, Medical University of Silesia in Katowice, Katowice, Poland; 2 Student Research Group at the Department of Cardiology, Faculty of Health Sciences in Katowice, Medical University of Silesia in Katowice, Katowice, Poland; 3 College of the Doctoral School, Faculty of Medical Sciences in Katowice, Medical University of Silesia in Katowice, Katowice, Poland; 4 Institute of Biology, Biotechnology and Environmental Protection, University of Silesia, Katowice, Poland

**Keywords:** knowledge, medical education, nicotine dependence, protection, students

## Abstract

**Background:**

The aim of the study was to assess the use of nicotine products, the level of nicotine dependence, and the circumstances of e-cigarette use among medical (physiotherapy) and non-medical students.

**Materials and methods:**

A cross-sectional survey was conducted among 388 students aged 18–29 from medical and non-medical universities in southern Poland. Data were collected in person via printed questionnaires administered to intact student groups during scheduled university classes. Nicotine dependence among e-cigarette users was assessed using the Fagerström Test adapted for vaping. Statistical analysis included descriptive statistics, the Chi-square test, and the Mann-Whitney U test.

**Results:**

Overall prevalence of nicotine use was similar in medical and non-medical students. Differences were mainly related to how participants consumed nicotine. Medical students more often used disposable and standard e-cigarettes, while traditional cigarettes were more common among non-medical students. Peer influence significantly affected nicotine use, and most e-cigarette use occurred in social situations.

**Conclusion:**

Medical education alone does not protect against nicotine use, but it appears to alter the preferred mode of nicotine intake. Preventive strategies should address social influences and increase awareness of risks associated with modern nicotine products.

## Introduction

In recent years, significant changes in nicotine use behaviors have been observed among young adults, and this trend is global (Palipudi et al., 2022). Traditional cigarette smoking is giving way to new forms of nicotine consumption, such as e-cigarettes, disposable e-cigarettes, and heated tobacco products (Albadrani et al., 2024; Sójka et al., 2024). The growing popularity of disposable e-cigarettes is particularly concerning. Particularly among youth and young adults, these devices are frequently perceived as a cleaner and less harmful alternative to traditional cigarettes. Colorful, flavored, and “safer” in appearance, they have captured the young adult market with a force unforeseen by healthcare professionals and legislators (World Health Organization Regional Office for Europe, 2025).

Beyond health concerns, the rapid proliferation of disposable e-cigarettes presents distinct environmental disposal challenges. These single-use devices contain non-biodegradable plastics, heavy metals, nicotine salts, and lithium-ion batteries, which can release toxic substances into soil and groundwater if improperly discarded (Boots et al., 2024; Turner et al., 2024). Although the lithium-ion batteries in disposable vapes are technically capable of over 700 recharge cycles, their single-use design leads to notable resource depletion (Reid et al., 2023). For instance, data from the UK indicate that up to 1.3 million disposable devices are discarded weekly, sending an estimated 10,000 kilograms of lithium to landfills annually (Askew, 2022; Material Focus, 2023). Paradoxically, a product promoted as a modern alternative to traditional cigarettes has thus emerged as a dual threat to both public health and environmental sustainability, underscoring the urgent need for targeted educational and regulatory initiatives among student populations (Ollila et al., 2024).

University students, across both medical and non-medical disciplines, frequently encounter an environment characterized by chronic academic stress, information overload, and psychological distress, including symptoms of anxiety and depression. These psychosocial factors significantly elevate the risk of initiating or maintaining nicotine product use, particularly e-cigarettes, as a maladaptive coping mechanism (Do et al., 2024; Do et al., 2025; Tasneem et al., 2025).

Although one might intuitively expect that medical student – having knowledge of physiology, pathophysiology, and the impact of stimulants on health – would be less likely to use nicotine products and be more aware of their long-term effects, empirical data do not always confirm this optimistic scenario. Medical students exhibit nicotine product use rates comparable to those of non-medical peers, showing that medical knowledge alone does not reliably translate into preventive health behaviors (Dilektasli et al., 2024). This raises the question: does medical education constitute a real protective barrier against nicotine dependence?

In this article, we not only compare the percentage of nicotine product users among medical and non-medical students, but also explore their choices regarding nicotine forms: from classic cigarettes, through standard e-cigarettes and heated tobacco products, to the fashionable and widely available disposable e-cigarettes.

## Aim of the study

The aim of the study was to assess the use of nicotine products, the degree of nicotine dependence, and the circumstances of using e-cigarettes by medical and non-medical students.

## Materials and methods

The research project was initiated after receiving a positive opinion from the Bioethics Committee of the Medical University of Silesia in Katowice (resolution no. BNW/NWN/0052/KB/252/24 of November 20, 2024).

The study group consisted of students from medical and non-medical universities located in southern Poland. Final statistical analysis included 388 correctly completed questionnaires, representing 97% of the 400 surveys distributed. Participants included 268 females and 120 males, with ages ranging from 18 to 29 years ($\bar{x}$ = 20.43 ± 1.50, where $\bar{x}$ represents the sample mean). Eligibility criteria for the study included studying a medical (physiotherapy) or non-medical (humanities) field of study and consent to participate.

In the context of research on nicotine dependence, it is crucial to precisely distinguish and define the terminology related to modern nicotine delivery systems.

A standard e-cigarette is an electronic vapor inhalation device designed for repeated use. It operates on a battery that requires regular charging. E-cigarettes utilize an e-liquid solution - typically composed of propylene glycol, glycerin, nicotine in varying concentrations, and flavoring agents - which is vaporized during use. Liquids are heated by the device’s heating element, creating an vapor, which is then inhaled by the user - a process known as vaping. Typical liquid packaging consists of 10 ml plastic containers, and the nicotine concentration ranges from 0 mg/ml to a maximum of 20 mg/ml, in accordance with applicable Polish law.

Unlike standard models, disposable e-cigarettes are designed for single use. Their design prevents recharging the battery or refilling the liquid. Once the pre-loaded liquid or battery is depleted, which typically occurs after about 700 puffs, the device becomes toxic waste requiring special disposal.

A heated tobacco product is an innovative device that operates by heating, rather than burning, tobacco. This device raises the temperature of the tobacco sufficiently to release an aerosol containing nicotine and other chemical compounds, without initiating the combustion process characteristic of traditional cigarettes. The goal of this technology is to reduce the user’s exposure to tar and other harmful byproducts of tobacco combustion by inhaling the aerosol instead of smoke. Like standard e-cigarettes, heated tobacco products require regular recharging. The source of nicotine in these devices are specially prepared, compressed tobacco sticks, sold in packs.

To ensure the uniformity and comprehensibility of the data, all of the above concepts were defined and explained in detail to the study participants before they began completing the research questionnaires. This minimized interpretation errors and increased the credibility of the collected information.

The full survey instrument comprised 99 items evaluating a broad spectrum of health behaviors, lifestyle factors, and psychological determinants among university students. To maintain thematic focus, the present study exclusively analyzes the subset of questions specifically addressing nicotine use prevalence, product preferences, and nicotine dependence levels. Data derived from the remaining questionnaire items address distinct research questions and are reserved for separate analysis in subsequent publications.

Participation was anonymous and voluntary. The questionnaire focused primarily on participants’ nicotine product use status and their primary sources of nicotine intake, such as disposable e-cigarettes, standard (reusable) e-cigarettes, heated tobacco products, and traditional cigarettes. General information (age, gender, place of residence, field of study) and data regarding the circumstances surrounding the participants’ initiation of nicotine were also collected. The survey also included questions about the situations in which respondents most frequently used nicotine products.

Physical nicotine dependence was evaluated using the Fagerström Test for Nicotine Dependence (FTND) which were specifically adapted by the authors to reflect the distinct operational characteristics of electronic cigarettes. The adapted version of the Fagerström test used in this study is based on the methodology established by Rahman et al. (2020). The FTND does not yield a binary positive/negative diagnostic cutoff; rather, it measures the degree of physical dependence on a continuous scale ranging from 0 to 10 points, categorized as low (0–3 points), moderate (4–6 points), or high (7–10 points). This questionnaire consisted of standard questions adapted for e-cigarette users. For example, instead of asking about the number of cigarettes smoked, the questionnaire asked about the number of vaping sessions.

The author’s questionnaire consisted of both single-choice and multiple-choice questions, with the option to add their own answers. In analyzing the results, particular attention was paid to the data from the multiple-choice questions. To avoid bias toward the first-choice answers, the order of response options was randomized across the questionnaires.

Paper questionnaires were distributed at universities during academic sessions, after obtaining consent from the heads of institutions and the participants themselves. In case of any doubts or misunderstandings, the study authors provided additional clarifications to ensure full understanding of the content and objective responses from participants.

Participants for this study were recruited using a personal, direct invitation method. No benefits were offered for completing the survey. Due to the lack of a centralized probabilistic sampling frame, a non-random convenience sampling methodology was employed based on institutional accessibility and willingness to cooperate. To mitigate intra-institutional selection bias, the sampling strategy targeted entire, intact student class cohorts during mandatory lecture hours.

The selection process aimed to obtain a diverse sample, which was achieved by inviting individuals from various locations and academic institutions - both medical (physiotherapy) and non-medical. Prior to distribution, researchers delivered a standardized terminology briefing to clarify specific product categories without referencing health risks.

Completed surveys, after being shuffled, were stored in a dedicated folder in a locked drawer, from which they were only removed when entering data into a spreadsheet. This ensured complete anonymity and prevented the identification of respondents.

All methods used in the study were in accordance with relevant guidelines and regulations. Data were collected and categorized in Microsoft Excel and then processed using descriptive statistics in Statistica 13.3. The Chi-square test was used for comparative analysis, and the Mann-Whitney U test was used for quantitative data that did not meet the criteria for a normal distribution (Shapiro-Wilk test *p* < 0.05). Multivariable logistic regression analysis was performed to evaluate the independent association between the field of study and e-cigarette use while adjusting for participant gender as a confounding variable. Several limitations of this study should be noted when interpreting the findings. First, the use of convenience sampling and voluntary participation introduces potential self-selection bias, which may have contributed to the relatively high prevalence of e-cigarette use observed compared to broader population estimates. Second, data were collected via self-reported questionnaires, exposing the findings to potential recall bias or social desirability bias, although anonymity was maintained to minimize this effect. Third, the cross-sectional study design precludes any causal inferences regarding the relationships between academic discipline, social factors, and nicotine dependence severity. Finally, because the study sample was restricted to specific student cohorts from selected faculties, external validity is limited, and findings may not fully represent the general population of young adults or students nationwide. Nonetheless, our results provide valuable comparative insights into product preferences and dependence patterns between medical and non-medical academic environments.

## Results

**1. General characteristics of the study group**

Table 1 presents the general characteristics of the study group, taking into account age, gender, place of residence and medical or non-medical education.

**TABLE 1:** General characteristics of the study group.

Variables		*n*	%
Study group n = 388			
Gender	Female	268	69.07%
	Male	120	30.93%
Age (years)	18–21	327	84.28%
	22–25	55	14.18%
	26–29	6	1.55%
Place of residence	Village	85	21.91%
	City	303	78.09%
Education	Medical	189	48.71%
	Non-medical	199	51.29%

n, number of participants.

The analysis covered 388 individuals. The majority of the study group were females (268; 69.07%). The dominant age group was 18- to 21-year-olds, constituting over 80% of the total (327; 84.28%). The vast majority of respondents lived in cities (303; 78.09%).

**2. Education and conditions of the respondents and their frequency of use of nicotine products**

Table 2 presents the influence of education on the respondents’ contact with disposable e-cigarettes, taking into account the division of the respondents into medical and non-medical students. Table 3 presents the influence of the environment (number of friends using nicotine-containing products) on the use of nicotine-containing products by the respondents. Table 4 presents the characteristics of the use of nicotine-containing products by the respondents.

**TABLE 2:** The influence of education on the respondents’ contacts with disposable e-cigarettes.

Variable	Have you ever used disposable e-cigarettes?				aOR	*p*
	Yes		No			
Education	*n*	%	*n*	%		
Study group n = 388 (100%)						
Medical students *n* = 199 (100%)	126	66.67%	63	33.33%	1.61	0.02136
Non-medical students *n* = 189 (100%)	110	55.28%	89	44.72%	1	
Gender	*n*	%	*n*	%	-	-
Female *n* = 268	167	62.31%	101	37.69%	1	0.37
Male *n* = 120	69	57.50%	51	42.50%	0.83	

n, number of participants; p, statistical significance; aOR, adjusted odds ratio.

**TABLE 3:** The influence of the environment (number of friends using nicotine-containing products) on the use of nicotine-containing products by the study participants.

Variable		Median percentage of friends who use nicotine-containing products	Lower quartile	Upper quartile	U-Mann Whitney test
Study group n = 388					
Use of products containing nicotine	Yes	40,00%	20,00%	70,00%	0,03365
	No	30,00%	5,00%	50,00%	
The entire study group	-	30,00%	10,00%	55,00%	-

n, number of participants.

**TABLE 4:** Characteristics of overall nicotine product use among participants.

Variable	The entire study group				*p*
Nicotine product use status	*n*		%		
Study group n = 388					
I have never used nicotine products	105		27.06%		-
I tried a nicotine product once and then stopped	102		26.29%		
I used to use nicotine products	74		19.07%		
I currently use nicotine products	107		27.58%		
**Variable**	**Education**				**p**
	**Medical (*n* = 189; 100%)**		**Non-medical (*n* = 199; 100%)**		
**Nicotine product use status**	***n***	**%**	***n***	**%**	
I have never used nicotine products	42	22.22%	63	31.66%	0.1875
I tried a nicotine product once and then stopped	54	28.57%	48	24.12%	
I used to use nicotine products	40	21.16%	34	17.09%	
I currently use nicotine products	53	28.04%	54	27.14%	

n, number of participants; p, statistical significance.

Medical students were more likely than non-medical students to have ever used disposable e-cigarettes (126; 66.67% and 110; 55.28%, respectively). After adjusting for gender, medical students remained significantly more likely to use e-cigarettes than non-medical students (aOR = 1.61, 95% confidence interval 1.07–2.43). Nicotine users had a significantly higher percentage of friends who also used these products compared to non-nicotine product users. In the study group, 107 (27.58%) respondents reported currently using nicotine-containing products. The proportion of active users of nicotine-containing products was similar in both groups (medical: 53; 28.04%, non-medical: 54; 27.14%). The key factor influencing nicotine product use status was the environment.

**3. Preferred nicotine products and education**

Table 5 presents the influence of education on the preferred nicotine-containing products among participants who declared regular use of such products (*n* = 121). The analysis includes disposable e-cigarettes, standard e-cigarettes, heated tobacco products, and conventional cigarettes, with respondents being permitted to select more than one product.

**TABLE 5:** The influence of education on the preference for nicotine-containing products.

Variable	Education				p
A product containing nicotine used:	Medical		Non-medical		
	*n*	%	*n*	%	
Study group n = 388					
Respondents declaring regular use of a specific nicotine-containing product (n = 121; 100%)					
Disposable e-cigarette	18	30.00%	15	24.59%	0.0267
Standard e-cigarette	27	45.00%	22	36.07%	
Heated tobacco product	12	20.00%	9	14.75%	
Cigarettes	3	5.00%	15	24.59%	

n, number of participants; p, statistical significance.

A statistically significant difference was observed in nicotine product preferences by field of study. A striking difference was observed in the use of traditional cigarettes, which were significantly more popular in the non-medical group (15; 24.59%) than in the medical group (3; 5.00%). Among nicotine product users, however, standard e-cigarettes were the most popular choice in both groups (27; 45.00% in the medical group and 22; 36.07% in the non-medical group).

**4. Circumstances of using disposable e-cigarettes**

Table 6 presents the characteristics of regular e-cigarette users (*n* = 82, encompassing users of both disposable and reusable/standard e-cigarettes), taking into account the circumstances of their use and first purchase.

**TABLE 6:** Characteristics of the studied group of students using e-cigarettes, taking into account the circumstances of their use.

In what situations do you most often reach for/buy/have you bought disposable e-cigarettes?	*n*	%
Study group (n = 388)		
E-cigarette users (n = 82; 100%)		
When I go/am at a party	67	81.71%
When I see my friends	17	20.73%
When I’m stressed	14	17.07%
When I go on holiday	10	12.20%
I use it constantly, regardless of the situation	7	8.54%
I usually use nicotine products alone	3	3.66%
At work	2	2.44%
**I bought my first disposable e-cigarette**	**n**	**%**
For the party	52	63.41%
Out of curiosity	25	30.49%
For social purposes	19	23.17%
Without any opportunity	14	17.07%
On a holiday trip	13	15.85%
On the recommendation of a friend	13	15.85%
When trying to quit smoking traditional cigarettes	10	12.20%

n, number of participants.

The main reason for using e-cigarettes was socializing. The vast majority (67; 81.71%) used them while going to a party. This is consistent with the circumstances of initiation – as many as 52 (63.41%) of the respondents bought their first disposable e-cigarette for a social gathering. Other popular reasons for first purchase included curiosity (25; 30.49%) and the desire to try it for social purposes (19; 23.17%).

**5. Level of nicotine dependence among e-cigarette users**

Table 7 presents nicotine dependence scores assessed via the adapted Fagerström Test for Nicotine Dependence. This table targeted regular e-cigarette users identified in Table 6 (*n* = 82). The difference in sample size (*n* = 67 vs. *n* = 82) reflects the presence of multiple-product nicotine use among some participants. To maintain assessment validity, the adapted Fagerström Test for Nicotine Dependence was completed exclusively for a single, primary source of nicotine—specifically, the product used most frequently by each participant. Table 8 presents number of nicotine products used by participants.

**TABLE 7:** Characteristics of the studied group of students using e-cigarettes, taking into account the degree of their nicotine dependence.

Variable	*n*		%		*p*
Study group (n = 67; 100%)					
Fagerström test result					
From 0 to 3	31		46.27%		-
From 4 to 6	23		34.33%		
From 7 to 10	13		19.40%		
**Variable**	**Medical (*n* = 36; 100%)**		**Non-medical (*n* = 31; 100%)**		**-**
	***n***	**%**	***n***	**%**	
Fagerström test result divided into medical and non-medical					
From 0 to 3	17	47.22%	14	45.16%	0.4261
From 4 to 6	14	38.89%	9	29.03%	
From 7 to 10	5	13.89%	8	25.81%	

n, number of participants, p, statistical significance.

**TABLE 8:** Number of nicotine products used by participants.

Number of nicotine products consumed daily	*n*	%
Study group n = 388		
1	88	22.68%
2	7	1.80%
3	5	1.29%
4	1	0.26%
I don’t use nicotine containing products	281	72.42%
Cigarette smokers n = 18		
People who smoke cigarettes only	10	55.56%
Dual nicotine products users (cigarettes + other nicotine containing product)	8	44.44%

n, number of participants.

Based on the Fagerström test, in the group of 67 (100%) e-cigarette users, the majority (31; 46.27%) demonstrated a low level of nicotine dependence (score 0–3 points). A high level of dependence (7–10 points) was found in 13 (19.40%) of the studied students. In the group of non-medical students, the percentage of individuals with a high level of dependence was higher than in the medical group (8; 25.81% and 5; 13.89%), however, this difference was not statistically significant. Table 8 presents the distribution of nicotine product usage among a study group of 388 participants. The majority of respondents (*n* = 281; 72.42%) reported not using any nicotine-containing products. Among those who do consume nicotine, most use a single product (*n* = 88; 22.68%), while a small proportion use two (*n* = 7; 1.80%), three (*n* = 5; 1.29%), or four products (*n* = 1; 0.26%). Among the 18 participants who reported cigarette smoking, 10 (55.56%) smoked cigarettes exclusively, whereas 8 (44.44%) concurrently used at least one other nicotine-containing product, consistent with the definition of dual use.

## Discussion

The results of the study presented a picture of nicotine use patterns among university students. Despite the assumption that medical students – due to their knowledge of health – would be less likely to use nicotine products, the overall prevalence of using nicotine products was similar between this group and students of non-medical humanities programs. Differences were mainly related to how participants consumed nicotine. This suggests that theoretical knowledge alone does not necessarily translate into refraining from nicotine use, as it is heavily outweighed by complex social dynamics and peer-related factors. Although they demonstrate a higher knowledge of the harmful effects of using nicotine products (Al-Sawalha et al., 2021), they are also at risk of developing nicotine dependence. The mechanisms that promote and prevent nicotine dependence are multidimensional, influenced by both education and its type, as well as social, demographic, and cultural factors (Han and Son, 2022). For example, an international study conducted among medical students and physicians in training in Brazil, the USA, and India revealed a high prevalence of e-cigarette use in Brazil and the USA, in contrast to a low prevalence in India. These results highlight the importance of cultural and regional context in nicotine use behavior (Degani-Costa et al., 2023).

The key factor influencing nicotine product use status in our study was the immediate environment. Nicotine users were significantly more likely to report that their friends also used nicotine products, consistent with the concept of social modeling, which posits that peer behavior plays a significant role in shaping individual habits (Prabhakaran et al., 2022). Numerous studies highlight the significant influence of peers on nicotine product use behavior among college students (Ismaniar et al., 2025; Singampalli et al., 2025; Thangeswaran et al., 2025). This phenomenon, widely documented in the scientific literature, suggests that preventive strategies should focus not only on the individual but also on their social environment to achieve lasting changes in health-related behaviors.

Interestingly, despite the lack of differences in the overall frequency of nicotine use, significant differences were observed in the preferred forms of nicotine consumption. Medical students more often than non-medical students reported using newer nicotine products, including disposable e-cigarettes. Harm perceptions surrounding e-cigarettes, however, appear to be increasingly complex. Although perceiving e-cigarettes as less harmful than combustible cigarettes has been associated with e-cigarette use, particularly among users and young adults, recent evidence also indicates a growing tendency to perceive vaping as equally or even more harmful than cigarette smoking. A recent systematic review demonstrated substantial heterogeneity in relative harm perceptions and highlighted that perceptions may change over time and in response to public health messaging. Therefore, the greater use of e-cigarettes observed among medical students in our study should not be interpreted as evidence that these students necessarily perceive e-cigarettes as less harmful than conventional cigarettes, (East et al., 2026; Tran et al., 2025; Vilcassim et al., 2025). Conventional cigarettes, on the other hand, were significantly more frequently chosen by non-medical students. These observations differ from data obtained in a study of Chinese students, where, although smoking various nicotine products was more common among non-medical students, no difference was observed in the choice of cigarettes or e-cigarettes between students of different fields (Song et al., 2023). This may be due to restrictions introduced in China on the electronic cigarette market (Tobacco Insider, 2023).

Disposable e-cigarettes, characterized by their attractive appearance and wide range of flavors, proved particularly popular in social settings. Most of the students surveyed used them at parties, and their first use often occurred in such settings. This phenomenon is confirmed by data from a study conducted in Australia, in which students from Melbourne were also most likely to use nicotine products in social settings (Tasneem et al., 2025). Medical students at Taibah University were also more likely to report using nicotine products if their friends or roommates smoked (Alzalabani and Eltaher, 2020). This demonstrates that disposable e-cigarettes function not only as a nicotine carrier but also as a socialization tool, potentially increasing the risk of their use among young people, for whom social contact is so crucial. Peer pressure and the desire to belong to a group play a key role in the dissemination of these products, making social environments an important target for future preventive interventions.

Analysis of nicotine dependence levels using the Fagerström Test revealed that the largest percentage of nicotine-using students had a low level of nicotine dependence (0–3 points), which may be related to occasional social use of e-cigarettes. However, it is worth noting that as many as 13 (19.4%) of the participants achieved scores indicating a high level of nicotine dependence. Although differences between fields of study were not statistically significant, it is alarming that high levels of nicotine dependence also occurred among those with medical knowledge. This demonstrates that nicotine dependence can develop regardless of education and health awareness. Currently, there are limited studies directly comparing e-cigarette dependence levels among medical and non-medical students, and to our knowledge, this is the only study comparing e-cigarette dependence using the Fagerström Test adapted for these subjects. Available research suggests that risk perception and nicotine dependence levels may differ depending on the field of study, but further research directly comparing these groups is necessary. Analysis of e-cigarette dependence levels among Jazan University students revealed a predominance of low-severity nicotine dependence, similar to our own study, which may suggest that some young adults use these products on an occasional or experimental basis. However, the presence of individuals with a higher degree of nicotine dependence indicates the need for educational and preventive measures before the nicotine dependence deepens and becomes permanent (Aqeeli et al., 2024).

The results of this study clearly indicate that both social factors and the attractive appearance of nicotine products have a significant impact on student behavior. Particular attention should be paid to the growing popularity of disposable e-cigarettes, whose “innocent” image may lead to downplaying the risk. A product intended to combat nicotine dependence to traditional cigarettes among adults is now a significant source of the growing wave of dependence among young people. While public health perspectives vary regarding the balance between adult smoking cessation and youth protection, preventing nicotine dependence among young populations remains a critical priority (Magalhaes et al., 2025). Therefore, it is necessary to consider tighter legislation regulating the availability of e-cigarettes, especially among young adults.

Our study demonstrates the need to implement more targeted preventive measures at the university level - not only among non-medical students but also among those who will be future health educators. Information campaigns should consider not only the health effects of using nicotine products but also the social and psychological context that fosters initiation. Instead of assuming that knowledge equals resistance to nicotine dependence, prevention should be approached with greater realism and holistic solutions.

## Conclusion

Medical students use nicotine at a similar rate to non-medical (humanities) students, although using different products. The similar level of nicotine dependence in both groups suggests that health knowledge alone is not always enough to refrain from risky behavior. Disposable e-cigarettes are often used in social situations, making them a socialization tool and pointing to the key role of peer influence in their dissemination. Specific educational and preventive strategies are needed that include raising awareness of the risks associated with new nicotine products and highlighting the influence of the social environment on their dissemination.

## Data Availability

The raw data supporting the conclusions of this article will be made available by the authors, without undue reservation after reasonable request.
